# Intercalary frozen autografts for reconstruction of bone defects following meta-/diaphyseal tumor resection at the extremities

**DOI:** 10.1186/s12891-022-05840-6

**Published:** 2022-09-30

**Authors:** Jingyan Yang, Wenze Li, Rongjie Feng, Dong Li

**Affiliations:** 1 Department of Pathology, The Second Hospital of Shandong University, Jinan, Shandong China; 2 Department of Orthopaedics, Shandong Provincial Hospital Affiliated to Shandong First Medical University, No. 324, Jingwu Road, 250021 Jinan, Shandong China

**Keywords:** Biological reconstruction, Limb salvage surgery, Liquid nitrogen, Malignant bone tumor, Recycling autograft

## Abstract

**Background:**

For patients with malignant limb tumors, salvage surgery can be achieved using endoprosthesis or biological reconstructions like allograft or autograft. In carefully selected patients, resected bone can be recycled after sterilization using methods like autoclaving, irradiation, pasteurization or freezing with liquid nitrogen. We evaluated the clinical outcome and complications of malignant limb tumors treated with intercalary resection and frozen autograft reconstruction.

**Methods:**

We reviewed 33 patients whose malignant bone tumors were treated by wide resection and reconstruction with recycling liquid nitrogen-treated autografts between 2006 and 2017. Limb function, bone union at the osteotomy site and complications were evaluated. Functional outcome was assessed using the Musculoskeletal Tumor Society (MSTS) scoring system.

**Results:**

The cohort comprised 16 males and 17 females, with a mean age of 35.4 years (14–76 years). The most common tumor was osteosarcoma (7 cases). Tumors were located in the humerus (5), ulna (1), femur (10) and tibia (17). The mean follow-up was 49.9 months (range 12–127 months). Of the 33 patients, 16 remained disease-free, and 3 were alive with disease. The mean size of the defect after tumor resection was 11.6 cm (range 6–25 cm). Bone union was achieved in 32 patients, with a mean union time of 8.8 months (range 4–18 months). Complications included 1 graft nonunion, 2 infections (1 superficial, 1 deep infection), 1 leg length discrepancy, 2 graft fractures and 3 local recurrences. The mean MSTS score was 87.2% (range 70–100%).

**Conclusion:**

Liquid nitrogen-treated tumor-bearing autograft is an effective option for biological reconstruction after meta-/diaphyseal tumor resection of long bones. This method has excellent clinical outcomes and is especially recommended for patients with no severe osteolytic bone tumors.

With the application of neoadjuvant chemotherapy and surgical staging, there has been great progress in the treatment of patients with malignant limb tumors [1, 2]. In recent years, limb salvage surgery has become increasingly popular after tumor resection because of its better overall survival rate and functional outcome than amputation [2, 3].

When the tumor is located in the diaphyseal or metaphyseal regions of long bones, intercalary excision is usually performed to remove the tumor and preserve the adjacent joints. The bone defect after tumor resection can be reconstructed with nonbiological (endo-prosthesis) [4] or biological (such as allograft [5], recycling tumor-bearing autograft [6], vascularized [7] or nonvascularized fibular graft [8], distraction osteogenesis [9]) reconstruction.

In recent years, reconstruction with recycling tumor-bearing autografts has become popular because it is adaptable and easily performed and has a very low risk of viral disease transmission [10]. Several methods of recycling have been developed, such as autoclaving [11], irradiation [12] and pasteurization [13]. However, these recycling methods require special equipment or strict thermal control and usually cause bone weakness, resulting in bone absorption and pathological fracture.

In 1999, based on in vitro and in vivo studies of the hypothermic effects of liquid nitrogen on osteosarcoma cells [14], Tsuchiya et al. introduced and clinically applied a reconstruction technique with tumor-bearing autografts treated with liquid nitrogen, and the long-term outcome was satisfactory [6, 15]. This technique has many advantages: simplicity, a perfect fit, a shorter union period, sufficient biomechanical strength, osteoinduction, osteoconduction, ease of soft-tissue attachment, low cost and a low risk of viral disease transmission.

In this study, we reviewed and analyzed the clinical outcome and complications of patients treated with intercalary frozen autografts after resection of malignant limb tumors.

## Methods

From 2006 to 2017, 40 patients with malignant bone tumors of the limbs underwent intercalary tumor resection and reconstruction with recycled frozen autografts. All patients were selected as following criteria: (1) Lesions were located in the diaphyseal or metaphyseal region of a long bone, and adjacent joints were preserved after tumor resection. (2) Age ≥ 14 years. (3) Lesions were widely excised with at least 2-cm margin. (4) Postoperative follow-up was at least 12 months, with clinical and radiological assessments performed. Of the 40 patients, 33 were eligible for inclusion in this study (Table 1). Seven cases were excluded from this study owing to younger than 14 years (5 cases) or inadequate resection margin (2 cases).

All diagnoses were confirmed by preoperative biopsy. Patients with osteosarcomas and Ewing’s sarcomas received neoadjuvant chemotherapy. All patients received postoperative chemotherapy, except for those with chondrosarcomas. The length of resection was determined based on preoperative Magnetic Resonance Imaging (MRI).

**Table 1:** Details of patients who underwent intercalary frozen autograft reconstruction

No.	Gender /age(yrs)	Site	Histology	Length (cm)	Fixation	Bony union (months)	MSTS score (%)	Complications (time;treatment)	Follow-up (months)	Outcome
1	F/54	tibia	lymphoma	7.5	nail	7	100	superficial infection (2 weeks po; debridement)	52	AWD
2	M/59	humerus	Metastasis (lung)	6	plate	10	93		45	AWD
3	F/21	femur	osteosarcoma	12	plate	8	83		118	NED
4	M/59	femur	malignant peripheral schwannoma	13	nail	6	90	recurrence (24 months po; resection)	32	DOD
5	F/16	tibia	osteosarcoma	10	nail	12	80		28	DOD
6	F/17	femur	osteosarcoma	15	plate	9	83		28	DOD
7	M/16	tibia	Ewing’s sarcoma	8	plate	4	93		54	NED
8	F/19	humerus	malignant peripheral schwannoma	12.5	nail	6	90	recurrence (30 months po; amputation)	44	NED
9	M/26	humerus	chondrosarcoma	10	plate	8	86		92	NED
10	M/19	tibia	Ewing’s sarcoma	11	nail	10	90		36	NED
11	F/51	humerus	chondrosarcoma	11.5	plate	10	93		88	NED
12	F/68	tibia	Metastasis (breast)	12	nail	12	86		12	DOD
13	M/14	tibia	Ewing’s sarcoma	8	plate	8	76	leg shortening, 4 cm (shoe lifts)	42	NED
14	F/15	tibia	malignant peripheral schwannoma	10	plate	8	83		60	NED
15	M/71	ulna	Metastasis (kidney)	9	plate	10	96		20	DOD
16	M/22	femur	osteosarcoma	25	plate	15	80	fracture (54 months po; prosthesis)	75	NED
17	M/70	femur	Metastasis (lung)	24	plate	-	86	nonunion (17 months po; die of disease)	17	DOD
18	F/49	femur	Metastasis (multiple Myeloma)	14.5	nail	8	86		127	AWD
19	F/14	tibia	osteosarcoma	12	plate	5	96		56	NED
20	F/32	tibia	synovial sarcoma	6	plate	6	93		53	DOD
21	M/55	femur	chondrosarcoma	15	nail	7	80	recurrence (36 months po; prosthesis)	40	NED
22	F/45	tibia	malignant fibrous histiocytoma	8	plate	6	93		36	DOD
23	M/16	femur	Ewing’s sarcoma	16	nail	8	90		32	DOD
24	M/16	femur	osteosarcoma	18	nail	10	93		50	DOD
25	M/17	tibia	chondrosarcoma	7.5	plate	11	86		63	NED
26	F/22	tibia	synovial sarcoma	13	plate	18	96		126	NED
27	M/56	humerus	malignant fibrous histiocytoma	11	nail	9	90		30	DOD
28	M/76	tibia	lymphoma	11	nail	8	70	deep infection (13 months po; amputation)	33	DOD
29	F/17	tibia	malignant fibrous histiocytoma	9.5	plate	10	90		48	NED
30	F/15	tibia	osteosarcoma	10	plate	10	70	Fracture (30 months po; internal fixation and allograft)	32	NED
31	M/31	tibia	fibrosarcoma	11	nail	6	86		14	NED
32	F/34	femur	synovial sarcoma	10	nail	5	86		42	DOD
33	F/55	tibia	Metastasis (lung)	7	nail	11	83		22	DOD

***Yrs*** years, ***M*** male, ***F*** female, ***MSTS*** musculoskeletal tumour society, ***po*** postoperation, ***AWD*** alive with disease, ***DOD*** died of disease, ***NED*** no evidence of disease

**Fig. 1:**
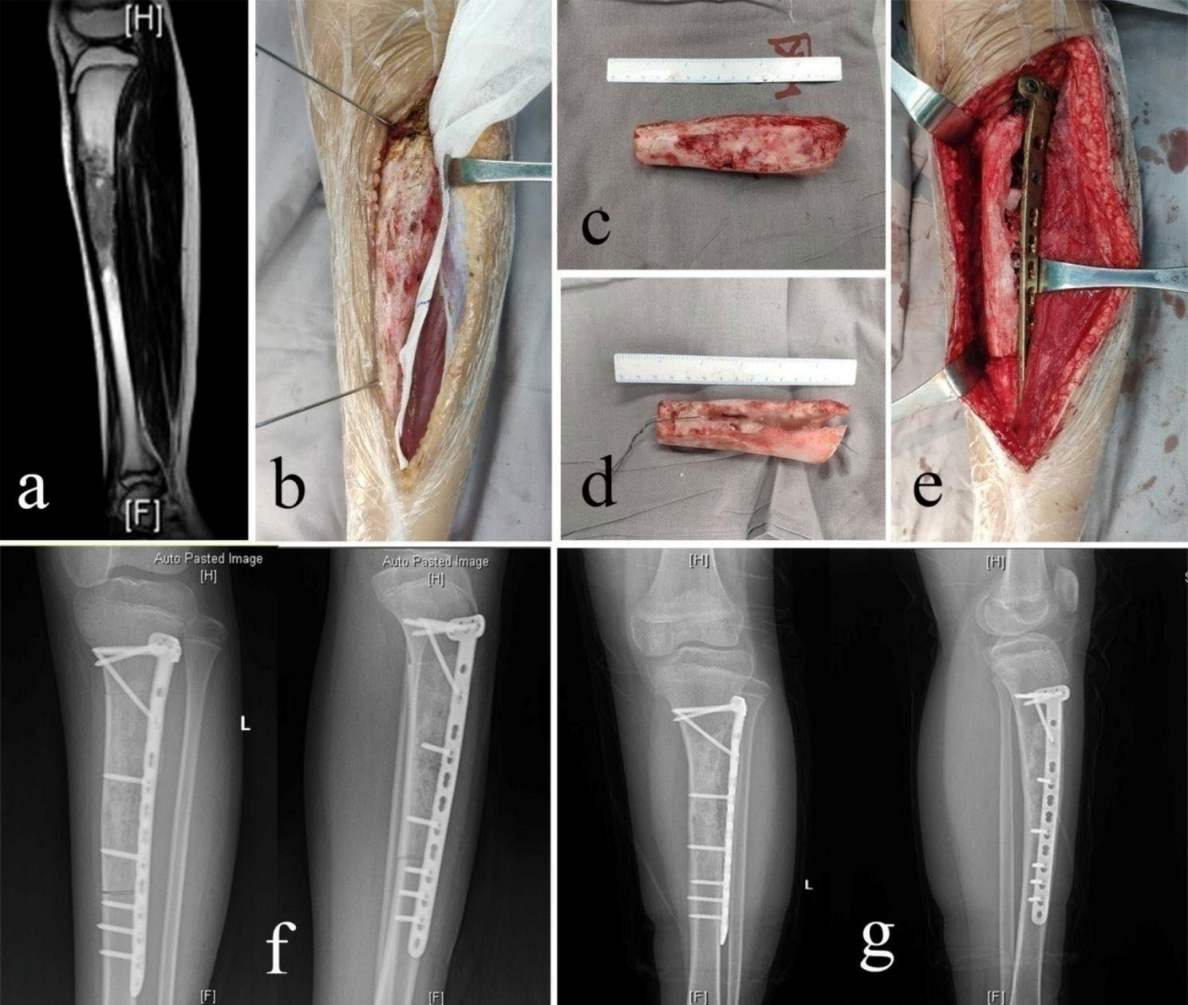
A 14-year-old female with osteosarcoma involving the diaphysis of the left tibia (case 19). (**a** Preoperative T2-weighted MRI (TR 2508.5ms, TE 100.0ms) showed a tumor located in the diaphysis of the left tibia. **b** Intercalary resection using a wire saw. **c** Resected bone. **d** Resected bone treated with liquid nitrogen. **e** The frozen autograft was reimplanted and fixed with a locking plate and screws. **f** Postoperative X-rays. **g** X-rays at 5 months after reimplantation showing union at both osteotomy sites.)

**Fig. 2:**
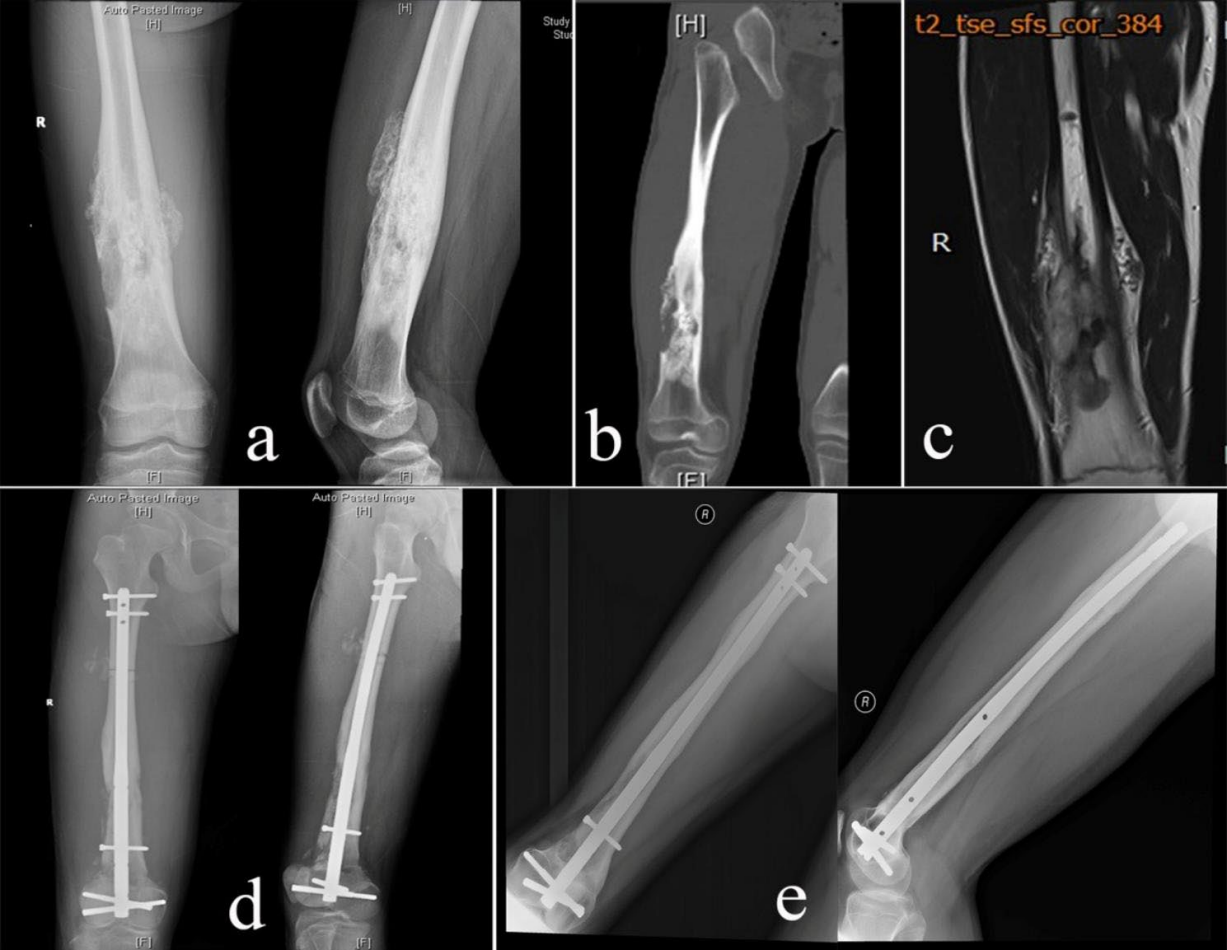
A 16-year-old male with osteosarcoma involving the diaphysis of the right femur (case 24). (**a, b and c** X-ray, CT and T2-weighted MRI (TR 3000.0ms, TE 98.0ms) showed the tumor involving the diaphysis of the right femur. **d** Postoperative X-rays after intercalary resection and recycling autograft reconstruction with intramedullary nail fixation. **e** X-ray at 10 months after reimplantation showing union at both osteotomy sites.)

The intercalary tumor-bearing autografts were treated with free freezing techniques using liquid nitrogen. Briefly, the tumor and a cuff of normal tissue peripheral to the tumor were excised en bloc, with a minimum 2 cm margin. The epiphyseal plate was sacrificed if necessary (in case 13, the tumor was 2-cm away from the epiphysis, transepiphyseal osteotomy was made to achieve wide resection). Intraoperative frozen sections of marrow/bone samples from osteotomy sites were taken to confirm the clean margin. Any attached soft tissues and gross tumors were removed from the resected bone, and the canals were curetted. Then, the bone was soaked in liquid nitrogen for 20 min, thawed at room temperature (~ 26 °C) for 15 min, and rinsed in distilled water for 15 min. The recycled bone was implanted into the bone defect and fixed by a locking plate (Fig. 1) or intramedullary nail (Fig. 2). Allografts or cement were used to fill bone defects as well as to increase strength after curettage in the recycled bone. Allografts were also performed at the junction sites.

Bone union was assessed with X-ray every month until the grafted bone united. Follow-up radiographs were obtained every 3 months for the first year and then every 6 months to check for recurrences and metastases.

Patients were encouraged to begin range of motion exercises immediately after the surgery. Partial weight bearing was allowed at one month after the operation. Full weight bearing was permitted until solid bone union was achieved. Radiological evidence of bone union in osteotomy sites included obscured osteotomy lines or sufficient bridging callus at the host-graft junction.

We evaluated the reconstruction methods, function of the affected extremities, bone union at the osteotomy site and complications. The functional outcome at the final follow-up was evaluated using the Musculoskeletal Tumor Society (MSTS) scoring system [16].

## Results

Details on the treatment results are presented in Table 1. The cohort comprised 16 males and 17 females, with a mean age of 35.4 years (14–76 years). The diagnoses were 7 osteosarcomas, 6 metastases, 4 Ewing’s sarcomas, 4 chondrosarcomas, 3 synovial sarcomas, 3 malignant fibrous histiocytomas of bone, 3 malignant peripheral schwannomas, 2 lymphomas, and 1 fibrosarcoma of bone. Out of 33 cases of tumors, 6 soft tissue sarcomas (3 synovial sarcomas and 3 malignant peripheral schwannomas) invade the bone; the rest tumors originate from the bone. Tumors were located in the humerus (5), ulna (1), femur (10) and tibia (17). This study was approved by the Institutional Ethics Committee of our hospital.

The mean follow-up was 49.9 months (range 12–127 months). At the last follow-up, 16 patients remained disease-free, 3 patients were alive with disease, and 14 patients had died of the disease or metastasis. The mean size of the defect after tumor resection was 11.6 cm (range 6–25 cm). During follow-up, the frozen autografts were removed in four cases (12.1%): two cases due to local recurrence, one due to severe infection and one due to fracture.

Bone union was achieved in 32 patients, with a mean union time of 8.8 months (range 4–18 months). Primary union (< 12 months) occurred in 28 of the 33 patients (84.8%), delayed union (≥ 12 months) in 4 (12.1%), and nonunion in 1 (3.0%). Fixation with intramedullary nailing was performed in 15 patients (45.5%); fixation with plating was carried out in the remaining 18 patients (54.5%).

Nine complications occurred in 9 patients, including 1 graft nonunion, 2 infections (1 superficial, 1 deep infection), 3 local recurrences, 2 graft fractures and 1 leg length discrepancy (LLD).

One 70-year-old male with femoral metastasis of lung cancer developed nonunion and died of the disease uneventfully (case 17). During the survival period, he only had slight limb pain and could walk on canes. No additional revision surgery was performed.

Infections developed in 2 patients with tibial lymphoma (case 1 and 28). In case 1, superficial infection occurred during the early postoperative period and was treated successfully with debridement. Case 28 was a 76-year-old male with severe diabetes; a deep infection occurred at 13 months after surgery and amputation was performed finally.

All three local recurrences occurred in the surrounding soft tissues (case 4, 8 and 21). Case 4 received another wide excision. In case 8, amputation was performed because of severe vascular involvement after recurrence. In case 21, malignant cells recurred very close to the autograft, invading the femur and were managed by wide resection and prosthesis reconstruction; the frozen autograft was removed.

Fractures occurred in two patients (case 16 and 30). One fracture (case 16) occurred owning to graft resorption and was managed by conversion to tumor prosthesis. The other autograft fracture (case 30) was seen at 30 months after surgery because of injury, and was successfully managed with internal fixation and allograft.

Leg length discrepancy (LLD) was seen in one 14-year-old teenager with tibial Ewing’s sarcoma (case 13). It was due to sacrifice of the epiphysis. The affected limb was about 4-cm shorter than the contralateral limb after follow-up of 42 months. He was managed conservatively using shoe lifts.

Functional outcomes were assessed with the MSTS scoring system, with a mean score of 87.2% (range 70–100%). The function of the limb was rated as excellent in 23 patients (69.7%), good in 7 (21.2%), and fair in 3 (9.1%).

## Discussion

There are several options to achieve reconstruction of intercalary meta-/diaphyseal defects following tumor resection. Segmental prosthesis has been widely used to provide good function with quick recovery; however, prosthesis’ durability becomes an issue over time [17, 18]. It cannot be considered a permanent solution for reconstruction. Distraction osteogenesis may be an alternative limb salvage technique, but it is time-consuming, unsuitable for large defects, and requires surgery many times [9]. Another choice of biological reconstruction is free vascularized or nonvascularized fibula graft; nevertheless, it has complications associated with the donor site; furthermore, the risk of stress fracture may be higher than other biological reconstructions [7, 8]. Many reports are mentioning the use of allograft can be a valuable solution to reconstruct the defect following segmental excision; however, mechanical complications are frequent, especially graft non-union and fracture; therefore, additional vascularized fibula graft is usually needed to ameliorate final results [5, 19].

Several techniques have been developed to devitalize and recycle the resected bone, including autoclaving, irradiation and pasteurization, but these methods demand strict thermal control and special equipment [11–13]. Furthermore, these techniques have been associated with a high rate of complications. Pan et al. used autoclaved autografts for limb-salvage reconstruction and reported that the rates of nonunion, infection, and fracture were 30%, 20%, and 20%, respectively [20]. Oike et al. reported a 14.8% infection rate and a 33% nonunion rate using extracorporeal irradiated autografts [21]. In a study investigating pasteurized autografts, the rates of deep infection, bone nonunion, and fracture were 15%, 42%, and 23%, respectively [22].

Liquid nitrogen has been used to treat bone tumors for some time. In 1969, Marcove et al. utilized liquid nitrogen for palliative treatment in patients with primary and metastatic bone tumors [23]. Many authors have reported the clinical use of liquid nitrogen as an adjuvant to curettage for benign, aggressive or malignant tumors [24–26]. In 2003, Yamamoto et al. verified the efficacy of exposing tumor cells to liquid nitrogen and found that the treated bone could maintain intact mechanical strength [14]. Tsuchiya et al. reported in 2005 clinical results of reconstruction with tumor-bearing bone treated with liquid nitrogen [6]. Since then, several authors have described the use of frozen autografts for reconstruction after excision of the tumor (Table 2) [6, 15, 27–30].

**Table 2:** Demographic data and outcomes of patients treated with frozen autograft in previous studies

Author Published year	Tsuchiya 2005	Tsuchiya 2010	Igarashi 2014	Paholpak 2015	Zekry 2017	Garg 2020
Cases	28	33	36	12	34	10
Follow-up (months) Mean (range)	28.1 (10-54)	30 (7-69)	101 (16-163)	Median 32 (12-86)	62 (24-214)	39.6 (6-97)
Rate of bony union	92.9%	93.9%	83.3%	100%	97.1%	93.8%
Union period (months) Mean (range)	6.7	7.3	6.2 (2-11)	8.2 (3-12)	10.2 (3-36)	5.2 (4-7)
Function (MSTS score) Mean (range)	71.4% Excellent 10.7%Good 10.7%Fair 3	75.7% Excellent 15.1%Good 9%Fair	72.2%Excellent 19.4%Good 8.3%Fair	79% (66.7-93.3%)	86.8% (30-100%)	80.2% (75-85%)
Complications						
Infection (NO./percentage)	3/10.7%	4/12.1%	4/11.1%	3/25%	2/5.9%	0
Recurrence (NO./percentage)	2/7.1%	3/9.1%	4/11.1%	1/8.3%	4/11.8%	0
Fracture(NO./percentage)	2/7.1%	3/9.1%	7/19.4%	1/8.3%	6/17.6%	0
Nonunion(NO./percentage)	2/7.1%	2/6.1%	6/16.7%	0	5/14.7%	1/10%

There have been many studies demonstrating the strong ability of liquid nitrogen treatment to kill tumor cells and the low possibility of local recurrence within recycled tumor-bearing autografts. The main mechanism of cryosterilization is crystal formation and dehydration of tumor cells, which are more sensitive to low temperatures than normal cells [31, 32]. The process of rapid freezing and slow thawing may cause fatal damage to tumor cells. It was also found that the number of cycles of liquid nitrogen treatment, either one or two, resulted in no significant difference with regard to effects of cell killing [14]. We used one-cycle steps of freezing in liquid nitrogen for 20 min, thawing at room temperature for 15 min and then rinsing in distilled water for 15 min. Only 3 local recurrences (9.1%) occurred in this cohort. The reported recurrence rate was approximately 7.1–11.8% (Table 2) [6, 15, 27–30]. Besides, most authors found recurrences were located in the surrounding soft tissues rather than the recycled bones, which was consistent with our results [6, 15, 29, 30]. Tsuchiya et al. attributed it to insufficient surgical resection [6]. We found that all three recurrences were near the side of the neurovascular sheath, probably owing to local satellite lesions or venous tumor thrombi. Special attention should be focused on the curative wide margin of this area.

The osteoinductive and osteoconductive properties of frozen autografts could be retained [33]. Histological sections showed newly formed bone, osteocytes and microvessels in all portions of the grafted bone removed from patients, and good bone fusion was seen at the host-graft junction [34]. The reported bony union rate was between 83.3% and 100%, with typically 5.2 to 10.2 months to reunion (Table 2) [6, 15, 27–30]. Compared with other reconstruction techniques, the union rates of frozen autografts are comparable with those of vascularized fibular grafts (86-100%) [7, 35], but higher than those of allografts (57-94%) [36–38] and nonvascularized fibular grafts (76-89%) [8, 35, 39]. In our study, bone union was seen in 32 of 33 patients (97.0%), with a mean union period of 8.7 months. Nonunion was seen in only one patient with femoral metastasis. The reported union rates of frozen autografts are also higher than those of other devitalized techniques including irradiated autografts (69-93%) [12, 21], autoclaved autografts (70%) [20], and pasteurized autografts (58-76%) [13, 22]. The high union rate may be attributed to the good preservation of bone morphogenetic activity of the frozen autografts.

As tumor patients survive longer, there are more concerns about autograft fracture. Reported fracture rates of frozen autografts range from 7.1 to 19.4% (Table 2) [6, 15, 27–30], which are lower than those of allografts (7-45%) [36–38], autoclaved bones (8.7-20%) [11, 20] and pasteurized bones (9.5-23%) [13, 22]. The rate of fracture is low because of early bony union and the intact initial strength of the recycled bone. Biomechanical tests have revealed that the initial strength of liquid nitrogen-treated bones is higher than that of autoclaved bones [14]. In addition, bone remodeling and revitalization in frozen bones occurs earlier than that in allograft, autoclaved or pasteurized bone [34]. Fracture thus remains a remote possibility, and only two (6.1%) occurred in our group. Nevertheless, in most patients, bone cortices are usually destroyed by malignant tumors, and the biomechanical strength might decrease depending on the extent of destruction. Therefore, a careful assessment of bone quality is needed before the resected bone is recycled. It is best for mild osteolytic or osteoblastic tumors but not for severe osteolytic lesions.

For frozen autograft reconstruction, there is no clear evidence that the type of fixation is related to the risk of non-union and graft fracture. To date, the selection of fixation type is mainly based on the surgeon’s preference. Intramedullary nail is easy to align correctly at diaphysis but difficult to control cortical displacement at metaphysis. Therefore, we preferred to use intramedullary nail if both osteotomy lines were at the diaphysis, while use plate for reconstruction if the osteotomy was performed through the metaphysis.

In addition to bone nonunion and fracture, other complications, such as infection and limb shortening, have been documented. In the current cohort, infection was seen in two patients (6.1%). The reported infection rates of frozen autografts range 5.9-12.1% [6, 15, 27, 29, 30], which is lower than those of allografts (0–28%) [36–38], irradiated autografts (14.8%) [21], autoclaved autografts (20%) [20] and pasteurized autografts (15%) [22]. Additional surgeries are usually needed to control infection, including debridement and irrigation or removal of the autograft. Garg et al. suggest that vancomycin-mixed normal saline be used to prevent infection during the recycling process [27].

Leg length discrepancy (LLD) is inevitable in adolescent patients, especially when the epiphysis is invaded by the tumor or sacrificed during resection [40]. To address LLD following limb salvage surgery, expandable prostheses have been developed [41]. However, besides the high complication rate, up to 50% of patients do not undergo further lengthening procedures due to oncological failures or overestimation of expected LLD [42]. Vogt et al. thought that the indication and the choice of length equalization method were always elective and decided by the informed patient; LLD between 2 and 5 cm could be equalized by shoe lift and/or insoles [43]. In skeletally immature patients, contralateral epiphysiodesis is an option to treat differences in leg length; but special attention should be paid to the effect of the procedure on body proportion and height [44]. Equalization of LLD of more than 5 cm can be achieved by distraction osteogenesis using external fixators or fully implantable intramedullary nails [45, 46]. In our cohort, the epiphysis of a 14-year-old teenager with tibial Ewing’s sarcoma was excised to achieve a negative margin. The predicted LLD was about 3 cm, and the patient was reluctant to receive contralateral epiphysiodesis. During follow-up of 42 months, the affected limb was about 4-cm shorter than the contralateral limb and was managed conservatively using shoe lifts. Additional limb lengthening may be considered after skeletal maturity.

This study included 6 cases of solitary bone metastasis. Patients with limb metastasis, especially those with severe osteolytic lesions, are usually treated with resection and segmental prosthesis reconstruction [47]. Segmental prosthesis currently gives the best short- and medium-term results for limb-salvage reconstruction in the treatment of bone metastases [20]. However, in developing countries, the cost of tumor prosthesis is beyond the reach of much of the population. Some authors reported the use of frozen tumor-bearing bone as an affordable alternative to the use of prostheses [6, 15, 29, 30]. Nevertheless, the technique should only be used in carefully selected patients with metastasis. We consider that frozen autograft is indicated for those patients if the lesion is solitary, the recycling bone has sufficient biomechanical strength, and good primary oncological control has been achieved.

The MSTS functional score has been reported to be 79–86.8% and “excellent” results were seen in most patients (over 70%) (Table 2) [6, 15, 27–30]. In our group, the mean MSTS score was 87.2%, and “excellent” results were seen in 69.7% of patients (23/33), with a good range of joint motion. The functional results are comparable with those reported in previous studies. With this reconstruction method, adjacent joints could be preserved, and recycled autografts could be easily reattached by soft tissues and muscles, which led to good muscle control and satisfactory functional scoring.

This study has some limitations. First, it is difficult to draw very objective conclusions because of the study’s retrospective nature, small sample size, and lack of a control group. Larger-scale controlled studies should be conducted. Second, this study includes a heterogeneous study sample with different diagnoses, tumor locations, and fixation methods. All of these factors make it difficult to analyze differences in outcome and complications. More studies are needed to contribute to standardizing this promising technique.

## Conclusion

Our results suggest that liquid nitrogen-treated tumor-bearing autograft is an effective option for biological reconstruction after meta-/diaphyseal tumor resection at the extremities. Once incorporated, this approach offers a permanent form of reconstruction with excellent clinical outcomes, including high functional scores and a low complication rate. The application of this method is especially recommended for patients with no severe osteolytic bone tumors.

## Data Availability

The datasets used and/or analyzed during the current study are available from the corresponding author on reasonable request.

## Abbreviations

MSTS: Musculoskeletal Tumor Society.
MRI: Magnetic Resonance Imaging.
LLD: Leg length discrepancy.
